# New Appliances and Things Medical

**Published:** 1900-04-21

**Authors:** 


### NEW APPLIANCES AND THINGS MEDICAL.

**[We shall be glad to receive, at our Office, 28 & 29, Southampton Street, Strand, London, W.C., from the manufacturers, specimens of all new preparations and appliances which may be brought out from time to time.]**

### DAHL'S STERILISED COW'S MILK.

(Herbert Francis and Co., 8, Arthur Street West, London, E.C.)

This milk differs entirely from condensed milk; it is nothing more than ordinary rich milk, which by a special process of sterilisation is rendered capable of remaining perfectly sweet for practically an indefinite period. The milk is absolutely free from living germs, and hence has advantages for the sick room and for domestic use which cannot be claimed for unsterilised fresh milk. The process of sterilisation does not affect the taste or flavour, and after briskly beating up the milk so as to mix in the cream, which has risen to the surface in the tin, it may be used for tea or coffee, and is indistinguishable from milk as usually met with. It is, however, questionable whether this milk can be used indefinitely for infant feeding—condensed milk, even though it contain the full amount of cream, cannot so be employed with impunity—it lacks what is called the antiscorbutic element, an element which is necessary for the proper nutrition of an infant, and which appears only to be contained in fresh vegetable and animal foods. It is doubtful whether sterilised milk which has been kept for any length of time possesses this property, even though it be scientifically and carefully preserved, as Dahl's excellent commodity undoubtedly is.

### SPRING BEDSTEADS.

(Isaac Chorlton and Co., 17, Blackfriars Street, Manchester.)

Chorlton's spring bedsteads possess a world-wide reputation, but, from the point of view of the reader of The Hospital, the special variety which is now being offered for infirmaries, schools, and other institutions will afford particular interest. These beds are immensely strong with patent rivetted rails, and patent "S" corners. The rails are made entirely without chilled castings, and therefore can stand any amount of rough usage; and further than that, the angles of the head and foot-piece are well rounded off, unlikely to harbour dirt, and easily kept clean. The frames are made flat for strength and delivered in one piece. The springs are of the chain mesh, or treble woven variety, with strengthening bands; they are exceedingly durable, and can be tightened at will.

